# Supplementary material for: The Impact of the COVID-19 Pandemic on Mental Health Care of Children and Adolescents in Switzerland: Results of a Survey among Mental Health Care Professionals after One Year of COVID-19
Source: Int J Environ Res Public Health. 2022 Mar 10;19(6):3252. doi: 10.3390/ijerph19063252 (PMC8953578; doi:10.3390/ijerph19063252)
Supplement: Supplementary file 1 [file ijerph-19-03252-s001.zip › ijerph-1572409-supplementary.pdf]

Anna Maria Werling, Susanne Walitza, Stephan Eliez, Renate Drechsler

Impact of the COVID-19 pandemic on mental health care of children and adolescents in Switzerland: Results of a survey among mental health care professionals after one year of COVID-19

Supplemental material

Table S1. If there was an increase in demand for treatment: How high was the increase at the time of greatest demand?

	N	%
About 20% more than usual	126	27.8
Up to 50% more than usual	150	33.0
About 50-100% more than usual	72	15.9
More than twice as much	41	9.0
No increase	18	4.0
Not stated	47	10.4

Table S2. What was the waiting time for admission to treatment before the pandemic?

	N	%
None / less than 1 month	122	26.8
1 to less than 2 months	126	27.7
2 to less than 3 months	59	12.9
3 to less than 6 months	59	12.9
More than 6 months	11	2.4
No waiting lists/other	14	3.1
Not stated	63	13.8
Total	454	100

Table S3. What is the current waiting time for admission to treatment (March/April 2021)?

	N	%
None / less than 1 month	50	10.9
1 to less than 2 months	61	13.4
2 to less than 3 months	50	11.0
3 to less than 6 months	137	30.1
More than 6 months	72	15.8
No waiting lists/ Admission freeze	27	5.9
Not stated	57	12.5
Total	454	100

Table S4. Did it happen that treatments could not be conducted as planned because of the pandemic? If yes, why? Summary of free-text comments (N=79)

	N
Therapy via telemental health	23
Telemental health not possible; rejected by patients, too difficult with children	5
Quarantine of patients or staff	11
COVID-19 rules/restriction of contact	9
Institution closed due to COVID-19	4
Focus on most urgent cases, sessions shortened, too many patients	11
Other	16

Table S5. What was or is particularly stressful/ burdensome in your work under pandemic conditions? Summary of free-text comments (N=112)

	**N**
High number of cases, difficult triage; desperate families who cannot be helped due to the lack of free capacity; no possibility to refer to child and adolescent psychiatry due to lack of therapy places	40
Increased organizational workload, impoverished team structures, staff shortages	10
Much more work taken on due to increased requests, but bordering on own overload	9
Own overload due to childcare under COVID-19, homeschooling	7
COVID-19 measures not always understandable; COVID-19 instructions often changing. Lack of prioritization of mental health during the pandemic and low appreciation of work for mentally ill children and adolescents; late vaccination of professionals in psychiatry, of professionals working with children	11
Own resources depleted because own recovery options also limited due to pandemic, general fatigue, uncertainty	7
Lack of financial coverage of online care at the beginning of the pandemic, lack of clarity especially for psychologists	5
Online therapy sessions at the beginning very helpful, later very tiring	4
Difficult to work wearing a mask with children	4
Lack of separation of professional and private life due to online sessions at home	4
General criticism of COVID-19 policy, COVID-19-related measures	3
Many last-minute cancellations from parents due to quarantine	3
Concern for own ill family members, team members	3
Concern about having to quarantine oneself and then not being able to work	2
Other	7

Table S6. Have there also been positive aspects of the pandemic for your work or for you personally?

	N	%
No positive aspects	147	32.4
Yes	280	61.7
Not stated	27	5.9
**If yes, please describe (summary of free-text comments) (N=280)**		
	N	
Familiarization with new techniques, experience with video therapy/ telemental health, easier to bring together all actors/family members via video session, possibility to carry out video conferences	128	
Concentration on the essential, deceleration (during lockdown)	45	
Benefits of working from home: no commute, quieter environment, more partnership in childcare, less travelling to meetings	35	
Online continuous education	28	
Core family moves closer together, more time with children	19	
Less stress during leisure time	17	
More conscious use of own resources	12	
More cohesion, within the family and among colleagues	10	
Better ecological balance, less noise	9	
Some patients have benefited	5	
Other	20	
